# Unexpected clipping failure of a full-thickness resection device during endoscopic full-thickness resection

**DOI:** 10.1016/j.igie.2024.08.006

**Published:** 2024-09-06

**Authors:** Yasutoshi Shiratori, Aaron Tokayer, Anthony Kalloo

**Affiliations:** 1 Department of Gastroenterology, Division of Gastroenterology, Maimonides Medical Center, Brooklyn, New York, USA; 2 Johns Hopkins University School of Medicine, Baltimore, Maryland, USA

Endoscopic full-thickness resection (EFTR) using a full-thickness resection device (FTRD; Ovesco Endoscopy AG, Tübingen, Germany) has emerged as an effective and safe technique for nonlifting colonic lesions, such as residual polyps after EMR.^1^ A high technical success rate has been reported.^2^, ^3^, ^4^, ^5^, ^6^, ^7^ However, reports of technical failure, such as misdeployment of clipping, are insufficient. Here we describe a case of FTRD clipping failure and our troubleshooting management.

## Case Description

**Figure 1:**
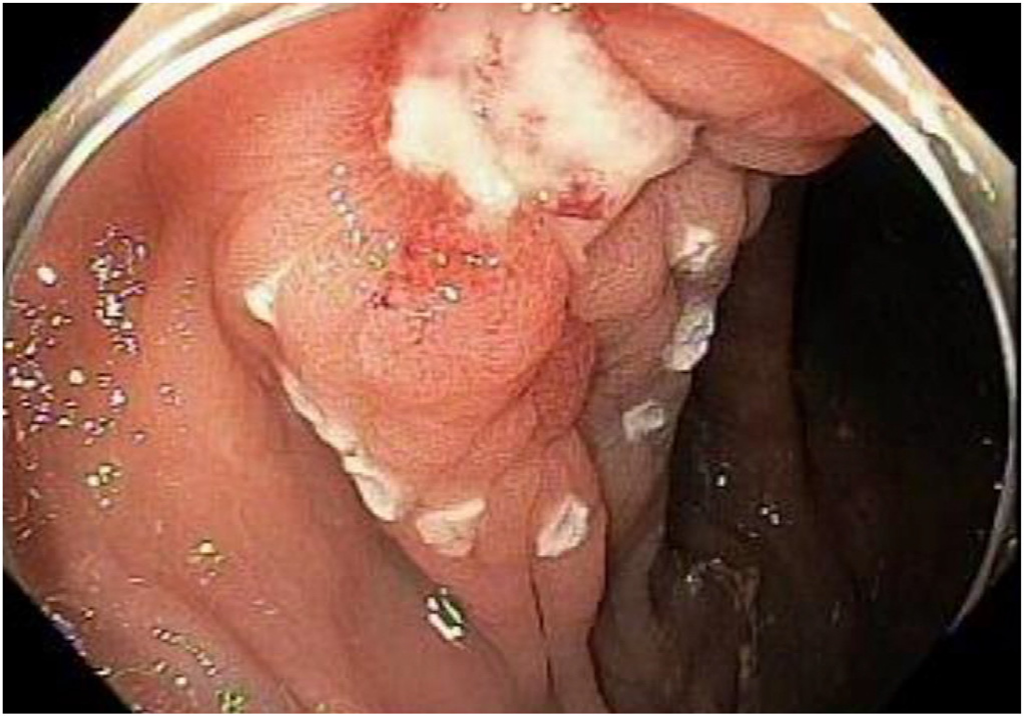
Residual colonic lesion after EMR, with the border marked using a coagulation probe. The lesion is located at the hepatic flexure.

A 74-year-old man was referred to our hospital to treat a residual polyp after EMR. The lesion was seen at the hepatic flexure with an EMR ulcer with significant scarring and adjacent tattoo (Fig. 1). Given the size and post-EMR condition, the decision was made to perform EFTR using an FTRD.

**Figure 2:**
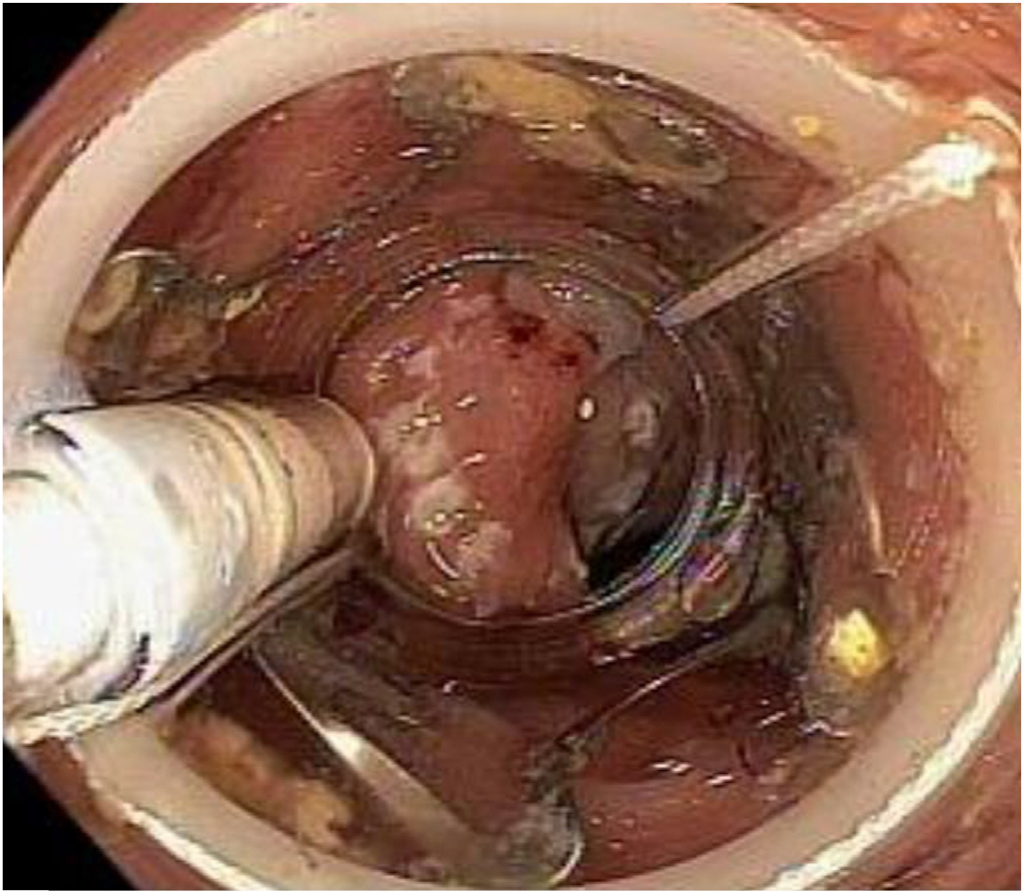
Endoscopic full-thickness resection using a full-thickness resection device.

**Figure 3:**
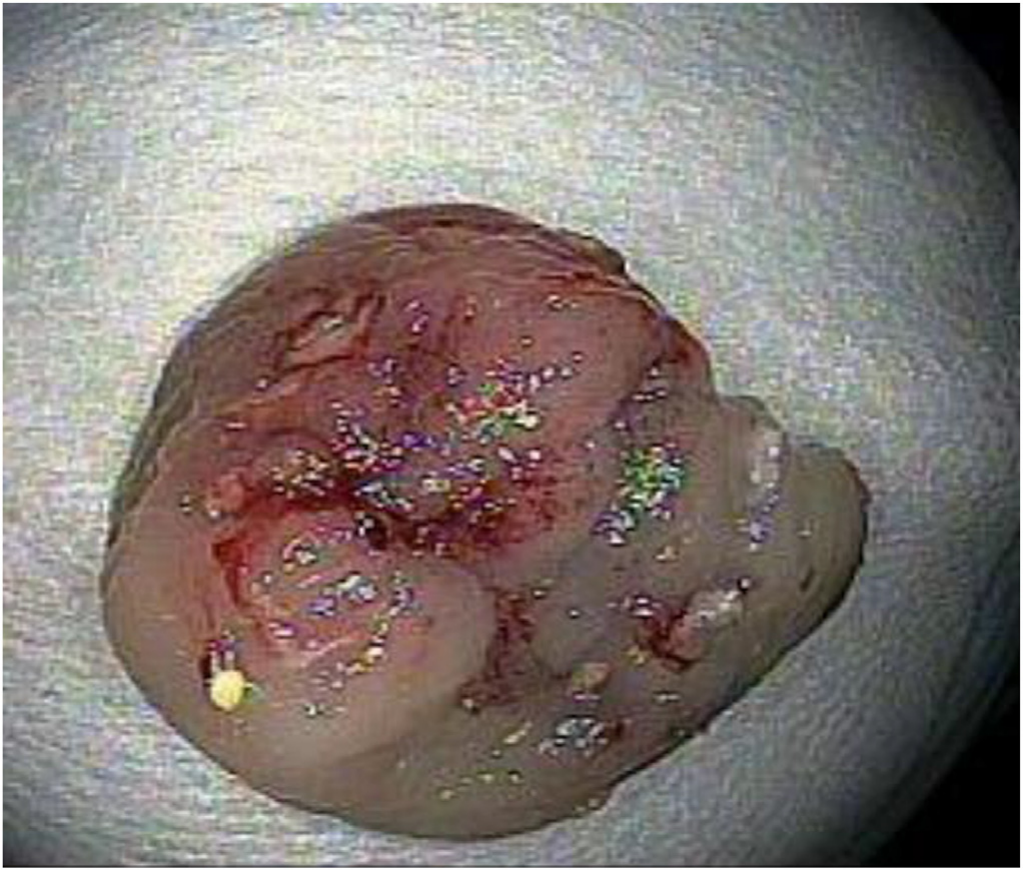
Resected colonic lesion with negative margins.

**Figure 4:**
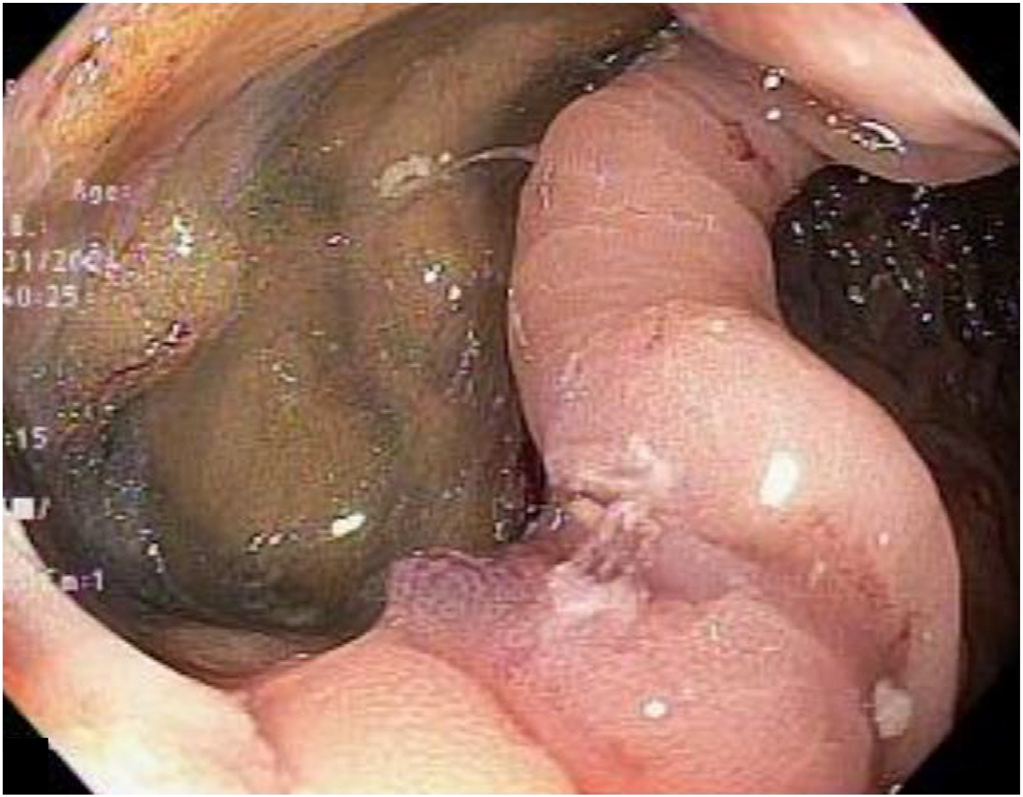
Colonic wall defect after clipping failure of the full-thickness resection device.

**Figure 5:**
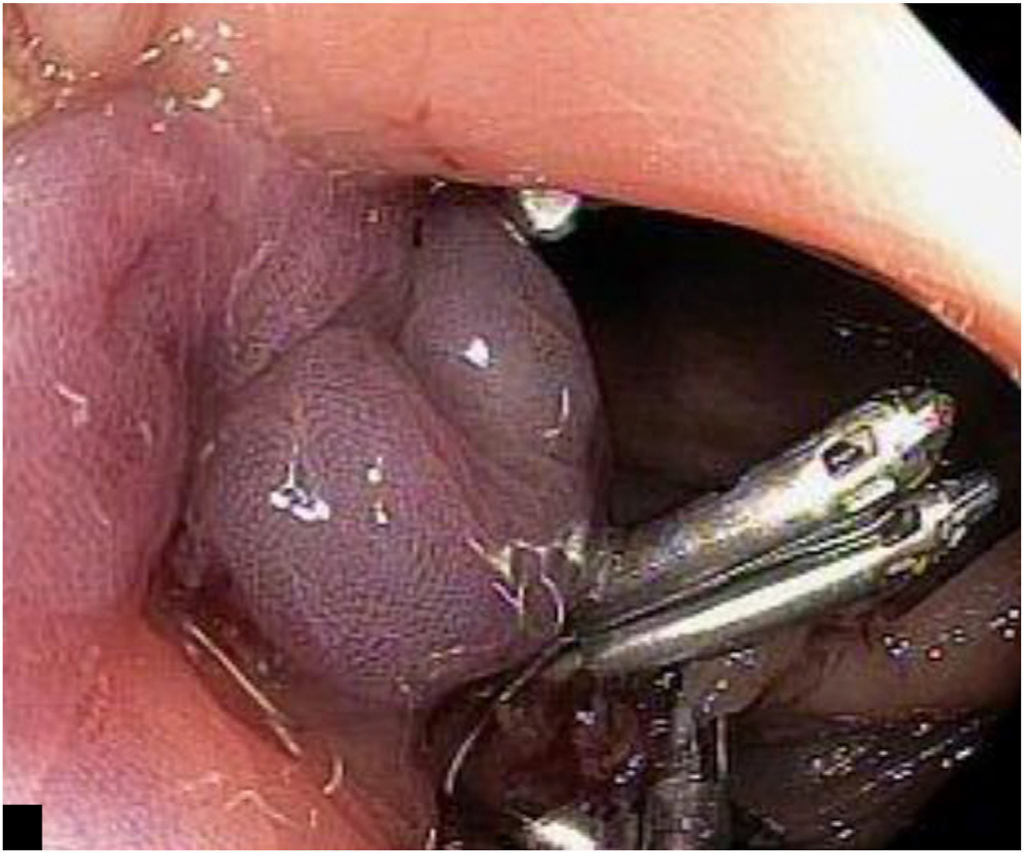
Closure using an over-the-scope clip and regular endoclips for the perforation.

The lesion was marked with a coagulation probe, and an applicator cap with a ready-to-use FTRD clip and integrated snare was attached. After pulling the lesion into the applicator cap using the grasper included in the products, the hand wheel for clipping was turned, and then the target tissue was cut with a snare (Fig. 2). The specimen was excised as planned (Fig. 3); however, the mounted FTRD clip, which should have been deployed before snare cutting, was not fully deployed. At the resected site, a 20 × 20-mm mucosal defect consistent with perforation was observed (Fig. 4). The defect was promptly closed with over-the-scope clips and 4 endoclips, which involved the omentum (Fig. 5).

The patient did not have any pain after the procedure and was discharged on postprocedure day 1 without adverse events. Final pathology showed a residual adenoma with complete excision with negative margins.

## Discussion

EFTR using an FTRD is a novel minimally invasive technique that allows the resection of nonlifting colorectal lesions, and a high R0 resection rate has been reported.^1^, ^2^, ^3^, ^4^, ^5^, ^6^ However, several systematic reviews have reported procedure-related adverse events such as postprocedure bleeding and appendicitis in 12% to 17% of cases.^2^^,^^3^ Perforation because of FTRD clipping failure during EFTR is very rare (.4%),^2^ but provisions should be made to treat a perforation in this eventuality.

A previous report treated this issue using over-the-scope clips, similar to our case, without surgical intervention. In our case, the operator, with >15 cases of FTRD, encountered a perforation. The acute angulation of the hepatic flexure and scope looping were believed to be the cause of clipping failure. Therefore, it may be necessary to straighten the scope when using the FTRD in a tortuous colon. In addition, both the operator and assistant must check for the movement of the white ring, which indicates successful clipping. For safer FTRD use, snaring should be avoided unless the white ring moves evenly with turning of the handle.

## Patient consent

The patient in this article has given written informed consent to publication of his case details.

## Disclosure

All authors disclosed no financial relationships.
